# Analysis of the epidemiological burden of acne vulgaris in China based on the data of global burden of disease 2019

**DOI:** 10.3389/fmed.2022.939584

**Published:** 2022-10-04

**Authors:** Yan Wang, ShengXiang Xiao, JianWen Ren, YanFei Zhang

**Affiliations:** Department of Dermatology, The Second Affiliated Hospital of Xi’an Jiaotong University, Xi’an, Shaanxi, China

**Keywords:** epidemiological, burden, acne vulgaris, China, GBD

## Abstract

Acne vulgaris is a chronic, inflammatory skin disease, which has brought an increasing disease burden to patients and society. But there is no systematic study on the disease burden and social development of acne vulgaris in China. This study aimed to analyze the epidemiological burden and trend of acne vulgaris in China from 1990 to 2019 based on the data in the global burden of disease 2019 (GBD 2019). The number of incidences/illnesses, age-standardized incidence/prevalence rates, disability-adjusted life years (DALYs), and DALY rate of acne vulgaris in China from 1990 to 2019 were obtained from the GBD 2019 to evaluate the epidemiological trends and age-period-cohort trends. The associations between disease burden and social development degrees were analyzed using a sociodemographic index. In 2019, the age-standardized prevalence and incidence of acne vulgaris in China were both at low levels in the world. From 1990 to 2019, the prevalent cases and incident cases of acne vulgaris in China rose firstly and then fell (peaked in 2005 and 2003, respectively), and the age-standardized prevalence/incidence/DALY rates showed growth trends continuously. The prevalence of acne vulgaris peaked in the 15–19 age group while the incidence peak age was 10–14 years old and there was an obvious gender difference, females were higher than males. With the increase of sociodemographic index (SDI) value, the morbidity of acne vulgaris showed a linear growth trend (*P* < 0.05). From 1990 to 2019, the disease burden of acne vulgaris is increasing in China, which is correlated with social and medical development. Active research on the epidemiological data of acne vulgaris and its relationship with the level of social development is important for both the diagnosis and treatment of acne vulgaris and for the development of health policies.

## Introduction

Acne vulgaris is a chronic, Inflammatory skin disease. Four factors have been thought to contribute to acne: hyper-secretion of sebum, abnormal proliferation and differentiation of keratinocytes in the hair follicle, bacterial colonization, and host inflammatory response (1). Acne occurs predominantly during adolescence and prepuberty and is one of the most common skin conditions. Globally, acne vulgaris was responsible for 4.96 million (95% CI 2.98–7.85) DALYs in 2019. Of these, 3.52 million (95% CI 2.11–5.64) DALYs occurred in 15–49 years old (2). In the 10–24 year-old group, acne was the 27th most common cause of rising DALYs in 1990 (Percentage of DALYs:1.1), rising to 19th in 2019 (Percentage of DALYs:1.6) (3). Acne can cause a substantial burden by negatively affecting the quality of life and mood of those affected, including an increased risk of anxiety, depression, and suicidal ideation.

The Global Burden of Disease (GBD) is developed by the Institute for Health Metrics and The University of Washington Evaluation (IHME) statistics and publishes data related to global diseases on its official website. Its latest databases have been updated to 2019, which are the most extensive and reliable databases on disease burden in the world at present (3). It provides a tool to quantify health loss from hundreds of diseases, injuries, and risk factors, so that health systems can be improved and disparities can be eliminated. The sociodemographic index (SDI) is a comprehensive indicator to measure the education level, per capita income and total fertility rate of a certain area. It is related to factors such as GDP per capita, the average number of years of schooling of the population over 15 years of age and the total fertility rate of the population under 25 years of age in the last 10 years. SDI ranges from 0 to 1, with lower values indicating a less developed region and higher values indicating a more developed region. SDI for countries and some provinces worldwide from 1990 to 2019 is published in an article by the official IHME team in the Lancet.

Due to the large population base in China, acne vulgaris has a large number of patients, which has brought a very serious disease burden to patients and society. Several studies have shown that the estimated prevalence of acne in China varies from 8.1 to 85.1%, depending on the study area and the age of the subjects (4–7). The disease burden of acne vulgaris is a serious situation, but there is no systematic study on the disease burden and social development of acne vulgaris in China. In this study, we used data from GBD 2019 to reveal the epidemiological burden of acne vulgaris over the past 30 years in China.

## Materials and methods

### Ethics

All protocols were approved by the Institutional Review Board of Health Science Center of Xi’an Jiaotong University, and performed according to guidelines governing ethics care in China. This study was performed in accordance with the rules laid down in the Declaration of Helsinki.

### Data sources

The data sources for this study were all published on the IHME website^1^ and searched using the GBD result tool. The diagnosis of acne vulgaris in GBD 2019 is based on the International Classification of Diseases, 10th Revision (ICD-10), with the code (ICD-10: L70. Excluding L70.4). In this study, prevalence, incidence, mortality, DALYs, and its corresponding age-standardized rates by world standard population of acne vulgaris were gathered by the Global Health Data Exchange query tool.^2^ The above indicators were analyzed, modeled, and estimated using the IHME Bayesian regression tool Dis Mod-MR 2.1, standardized for the world population and reported as age-standardized prevalence, incidence, and DALY rates per 100,000 persons. All estimates were generated with 95% confidence intervals (95% CI), including all uncertainties due to measurement error, bias and modeling. 95% CIs were taken from the 2.5th and 97.5th percentiles of the 1000th sample (3).

### Search method

The IHME website (see text footnote 1) was accessed from a browser and searched using the gbd-result-tool. Under the “Single” tab, single indicators were searched by selecting the location, age, year, and measure metric according to the project.

### Statistical analysis

Data such as Prevalent Cases, Age-standardized Prevalence Rate, Incident Cases, Age-standardized Incidence Rate, Disability-Adjusted Life Years (DALYs) and Age-standardized Disability-Adjusted Life Year Rate (DALY rate) were analyzed, the prevalence trend of acne vulgaris in China was evaluated by age, period, and birth cohort, as well as the relationship between the SDI and the prevalence of acne vulgaris and DALY rates in China, were analyzed. Office Excel software was applied to tabulate the data. As the GBD has standardized the age and given 95% CI, R3.6.2 software could be applied directly, SPSS 26.0 software was used, the matplotlib package was installed and loaded, statistical analysis was performed and plotted, and the partial correlation analysis was applied to analyze the correlation of SDI with age-standardized incidences and DALY. *P* < 0.05 was statistically significant.

## Results

### Basic information of acne vulgaris in China

Among the 204 countries and regions assessed by GBD 2019, China ranked 176th in both age-standardized prevalence and incidence of acne vulgaris, with prevalence and incidence levels higher than most Asian countries and lower than a few East Asian countries such as Japan and Korea. From 1990 to 2019, the prevalent and incident cases in China demonstrated a fluctuating upward trend and the prevalence and incidence increased continuously. As shown in Table 1, in 2019, the total number of illnesses of acne vulgaris in China was 42,793,474 (95% CI 39,101,682–46,784,200), of which 18,583,738 (95% CI 16,976,918–20,376,216) were males, accounting for 43.43%, and 24,209,736 were females (95% CI 22,125,383–26,444,134), taking up 56.57%. Compared with the total number of illnesses cases in 1990 (41,184,974 95% CI 36,959,671–46,045,011), it increased by 3.91%. The prevalence rate increased from 3,037.89 (95% CI 2,732.37–3,380.82)/100,000 in 1990 to 4,124.60 (95% CI 3,713.35, 4,571.75)/100,000 in 2019, with an increase rate of 35.77%. In 2019, the incident cases of acne vulgaris in China was 20,197,656 (95% CI 18,146,798–22,640,673), with an incidence rate of 2,086.69 (95% CI 1,837.00–2,382.25)/100,000 of which 8,782,836 (95% CI 7,923,728–9,797,091) were males, accounting for 43.48%, with a incidence rate of 1,691.33 (95% CI 1,497.61–1,921.37)/100,000 and 11,414,819 (95% CI 10,215,392–12,792,744) were females, occupied for 56.52%, and the incidence rate was 2,544.04 (95% CI 2,230.68–2,918.33)/100,000. The number of incident cases increased by 1.33% and the incidence rate increased by 34.13%.

**TABLE 1:** The prevalent cases/prevalence rates, incident cases/incidence rates, and disability-adjusted life years (DALYs)/DALY rates of acne vulgaris in China in 1990 and 2019.

	Year	Male	Female	Total
**Prevalent cases**	1990	17,166,395 (15,419,998–19,107,049)	24,018,580 (21,426,848–26,882,653)	41,184,974 (36,959,671–46,045,011)
	2019	18,583,738 (16,976,918–20,376,216)	24,209,736 (22,125,383–26,444,134)	42,793,474 (39,101,682–46,784,200)
	(%)	8.26	0.80	3.91
**Prevalence rates**	1990	2,450.05 (2,213.45–2,722.75)	3,662.90 (3,280.62–4,086.59)	3,037.89 (2,732.37–3,380.82)
	2019	3,366.05 (3,027.80–3,740.96)	4,989.51 (4,486.68–5,527.70)	4,124.60 (3,713.35–4,571.75)
	(%)	37.39	36.22	35.77
**Incidence cases**	1990	8,330,565 (7,438,891–9,345,903)	11,602,405 (10,247,978–13,213,393)	19,932,970 (17,718,779–22,559,110)
	2019	8,782,836 (7,923,728–9,797,091)	11,414,819 (10,215,392–12,792,744)	20,197,656 (18,146,798–22,640,673)
	(%)	5.43	−1.62	1.33
**Incidence rates**	1990	1,249.76 (1,111.09–1,411.82)	1,882.23 (1,654.34–2,166.95)	1,555.71 (1,375.71–1,775.96)
	2019	1,691.33 (1,497.61–1,921.37)	2,544.04 (2,230.68–2,918.33)	2,086.69 (1,837.00–2,382.25)
	(%)	35.33	35.16	34.13
**DALYs**	1990	371,097 (224,063–597,192)	516,480 (313,735–825,718)	887,577 (540,585–1,423,216)
	2019	401,293 (238,229–639,466)	520,500 (314,241–826,668)	921,793 (555,006–1,462,140)
	(%)	8.14	0.78	3.85
**DALY rates**	1990	52.95 (31.99–85.21)	78.77 (47.76–125.95)	65.46 (39.90–105.04)
	2019	72.83 (43.61–115.80)	107.64 (65.17–170.70)	89.09 (53.68–141.34)
	(%)	37.54	36.66	36.09

“%” means percentage change between 1990 and 2019 (%). The 95% confidence interval (CI) is in brackets.

### Prevalence trend of acne vulgaris in China

**FIGURE 1:**
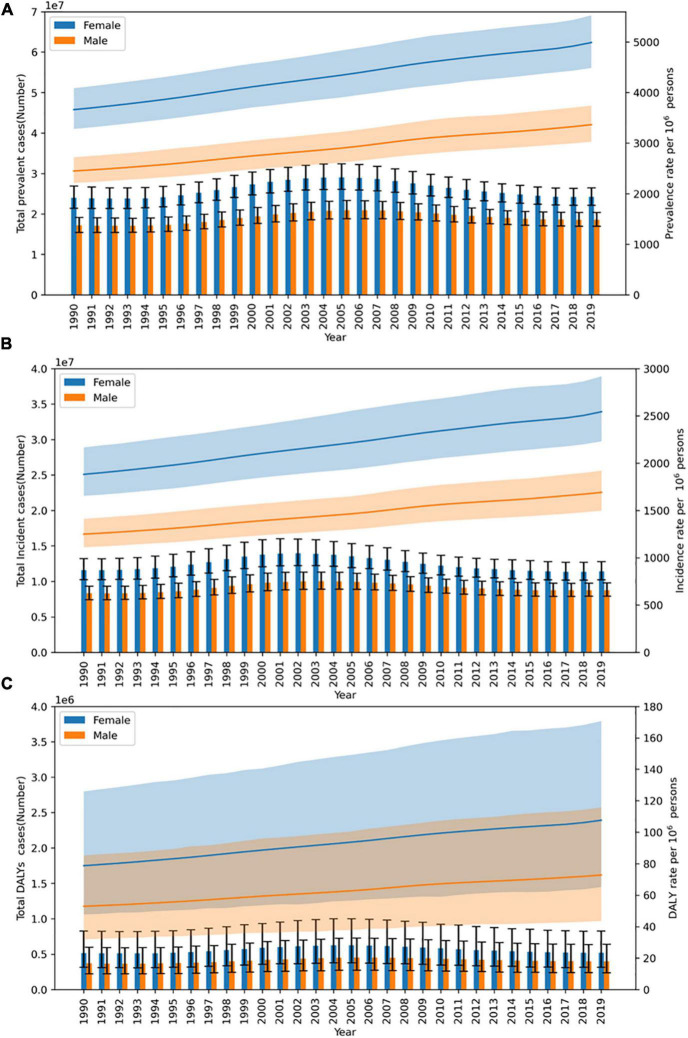
Trends of acne vulgaris in China from 1990 to 2019. **(A)** Prevalent cases and rate. **(B)** Incidence cases and rate. **(C)** Disability-adjusted life years (DALYs) cases and DALY rate.

**FIGURE 2:**
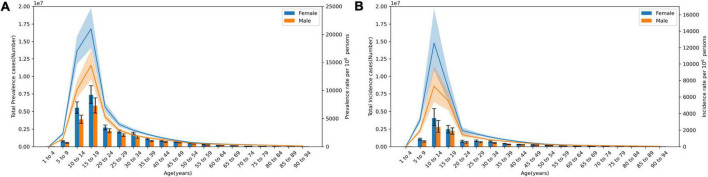
Trends of acne vulgaris with age in China at 2019. **(A)** Prevalence rate and cases. **(B)** Incidence cases and rate.

From 1990 to 2019, the prevalent cases and incident cases of acne vulgaris in China rose firstly and then fell (peaked in 2005 and 2003, respectively), which both showed a lower prevalence in males than in females. The age-standardized prevalence and incidence rates also showed an increasing trend and males were lower than females (Figures 1A,B). Disability-adjusted life years (DALYs) of acne vulgaris in China remained roughly unchanged from 1990 to 2019, while age-standardized DALY rates showed an increasing trend, with DALYs of 887,577 (95% CI 540,585–1,423,216) in 1990 and 921,793 (95% CI 555,006–1,462,140) in 2019, the increase rate was 3.86%. The age-standardized DALY rate increased from 65.46 (95% CI 39.90–105.04)/100,000 in 1990 to 89.09 (95% CI 53.68–141.34)/100,000 in 2019, an increase of 36.09% (Table 1 and Figure 1C). The data of 2019 suggested that the prevalence and incidence of acne vulgaris in China indicate some correlation with age. The prevalent cases showed a gradual increase with age, peaking at the 15–19 age group for both males [5,797,310 (95% CI 4,788,772–6,931,577)] and 7,359,734 (95% CI 8,668,363–6,156,506) for females, respectively, then followed by a decreasing trend with age. The prevalence rate also showed a similar trend (Figure 2A). The incidence cases and rates also suggested a trend of increasing firstly and then decreasing with age. Both males and females peaked at 10–14 age group in incident cases 2,799,100 (95% CI 2,062,632–3,731,282) for males and 4,059,147 (95% CI 2,989,338–5,401,943) for females, respectively (Figure 2B).

### Age, period, and birth cohort trends of acne vulgaris in China

Period trends in incidence rates showed that from 1990 to 2019, the incidence rate of acne vulgaris increased in all age groups. The slope of the line indicates the rate of increase, which varied between each age group.

**FIGURE 3:**
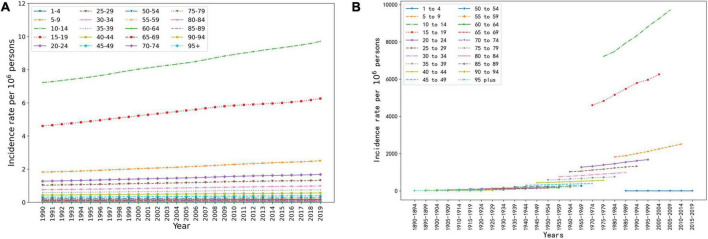
Age, period, and birth cohort trends of acne vulgaris in China. **(A)** The period trend of acne incidence rate in different age groups in China. **(B)** The birth cohort trend of acne incidence rate in different age groups in China.

It showed a more pronounced increase in the 5–24 years age group. During these 29 years, the incidence of acne vulgaris increased firstly and then decreased with the increase of age, while the age group that reached the peak incidence rate did not change with the increase of years (Figure 3A). The birth cohort trend of acne incidence rate showed that the effect of age on the incidence rate of acne vulgaris in China tends to increase and then decrease, which means the effects of both higher and lower ages on incidence were smaller. The post-2006 birth cohort showed partial outliers due to the limited age cohort. In contrast, the birth cohort for each age group between 10 and 19 years has a significantly higher effect on incidence than other age groups, which is shown in the graph by the slope of the lines being significantly larger than other groups (Figure 3B).

### Relationship between morbidity of acne vulgaris and sociodemographic index in China

**FIGURE 4:**
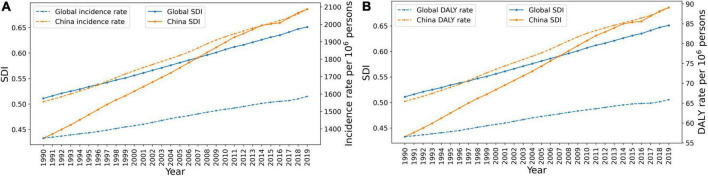
The relationship between acne vulgaris and sociodemographic index (SDI) in China and around the world. **(A)** The relationship between age-standardized incidence rate of acne and SDI. **(B)** The correlation between acne disability-adjusted life year (DALY) rate and SDI.

From 1990 to 2019, SDI values in China showed a continuous upward trend. As shown in Figure 4, both global and Chinese data suggested that the age-standardized incidence rate and DALY rates show an increasing trend as SDI increases. From 1990 to 2019, China’s SDI increased, being significantly lower than the global average in 1990, exceeding the global average from 2007 onward, and by 2019, China’s SDI was significantly higher than the global average. At the same time, the age-standardized incidence rate and DALY rate of acne vulgaris in China were significantly lower than the global average. The age-standardized incidence rate of acne in China was highly correlated with the SDI in China (*r* = 0.631, *P* < 0.05), the age-standardized incidence of acne globally was positively correlated with the SDI worldwide (*r* = 0.461, *P* < 0.05) (Figure 4A). Correlation analysis of the data suggested that the Chinese acne DALY rate was highly correlated with the Chinese SDI (*r* = 0.814, *P* < 0.05), the correlation between the global acne DALY rate and the global SDI was not statistically significant (*r* = −0.154, *P* > 0.05) (Figure 4B).

## Discussion

Acne vulgaris is a chronic inflammatory disease of the hair follicle sebaceous gland, characterized by papules, pustules, nodules, cysts, and scarring, often accompanied by seborrhea, and is a common skin disease in adolescence in dermatology clinics. According to the GBD study, the prevalence rate of acne is 9.4% (8), making it the eighth-most prevalent disease in the world (9). Approximately 85% of young people aged 12–25 years are affected by acne vulgaris, and the trend continues to increase in this population (9, 10). According to a survey on the epidemiological characteristics of adolescent acne in North East China, the total prevalence of adolescent acne was 51.30% (52.74% in males, 49.65% in females) (11). In addition, a growing body of epidemiological data suggests that acne also affects a significant number of adults (12) and that women are more likely to be affected than men (13–15).

The visible nature of acne, symptoms, and sequelae all contribute physically and psychosocially to the overall burden of disease, as do the costs required for management. The direct costs of acne include resources for prescription and over-the-counter medications, cosmetics, clinician visits, medical procedures, and hospital visits. Acne is recurrent and difficult to cure, so the total direct financial burden on people in the long term cannot be ignored. The indirect costs reflect changes in productivity, and acne, especially serious acne sequelae such as scarring, can have a significant impact on obtaining employment. And the intangible costs represent deficits in quality of life. Although it is difficult to quantify the effects of acne on psychological wellbeing and social functioning, preliminary research has identified problems with self-esteem/self-confidence, body image, social withdrawal, depression, anger, limitations in lifestyle, and other psychosocial function areas.

Treatment of acne can be done topically with retinoids, azelaic acid, benzoyl peroxide, or topical antibacterials or systemically with oral antibacterials, hormonal therapies, or isotretinoin. Light and laser treatments (photodynamic therapy, blue light, intense pulsed light) are a growing interest in new non-invasive therapies for acne. Among the direct costs, the most expensive agents were topical antibiotics, topical retinoids, and topical retinoid combination preparations. Oral antibiotics are less expensive and are widely used for the treatment of moderate to severe acne due to their antibacterial and anti-inflammatory activities. Due to the increasing number of resistant bacteria, Antibiotic resistance places a significant burden on patient health and healthcare systems. Long-term use of oral antibiotics remains a major problem, not only on the skin but in all body parts with resident commensal flora. According to a study, the society’s economic costs of antibiotic resistance are probably much greater than those of sick leave due to disease alone (16, 17).

Recent studies showed that the prevalence of acne vulgaris is increasing in almost all countries (18). In a study of skin and subcutaneous diseases in the Chinese population from 1990 to 2019, it was found that the increase of age-standardized prevalence rate was the highest for acne vulgaris (EAPC = 1.087, 95% UI = 1.052–1.110) (19). The prevalence of acne varies by country and ethnicity, and according to the GBD, in 2019 the age-standardized prevalence and incidence levels of acne vulgaris in China were much higher than in most Asian countries, and third in Asia after Korea and Japan, with data showing a positive correlation between acne burden and national wealth, suggesting that acne prevalence is lower in rural, non-industrialized areas than in modernized populations (20). Possible reasons for this include differential access to healthcare, differential socioeconomic status of patients, and cultural perceptions of skincare and beauty as well as dietary habits and lifestyle. In addition, underdiagnosis may contribute to the lower prevalence of acne in non-industrialized areas. The rapid pace of social development in China from 1990 to 2019 is remarkable to the world, and the prevalence and incidence rate of acne vulgaris has increased accordingly, a trend that is verified in the distribution of period trends across age groups. Meanwhile, the incidence rate of acne vulgaris has increased in different age groups, which may be related to changes in lifestyle and diet, increased psychological stress, and may also be associated with the improvement of diagnosis and changes in perceptions of skincare and beauty. From 1990 to 2019, the age-standardized DALY rate for acne vulgaris in China increased slowly, which is associated with increases in morbidity, population longevity, and a longer survival time with the disease due to advances in treatment.

Data from 2019 indicated that the onset of acne vulgaris is closely related to age, and although it can occur at all ages, the peak incidence age is concentrated in the 15–24 age group, and the age trends in different years further indicate similar characteristics in the age distribution of acne vulgaris. Although incident cases rose in all age groups as the years increased, the rate of increase varied and the peak age of incidence shifted back, which may be another indication of the increasing longevity of the population and the improvement in the overall health of the population.

In 2019, China (0.686) was assessed as an intermediate SDI level (0.6077–0.6896). The SDI level in China increased very rapidly from 1990 to 2019, significantly exceeding the world average by 2019, while the growth in the morbidity of acne vulgaris in China also exceeded the global growth level, suggesting that the incidence of acne vulgaris is closely related to SDI, reflecting on the one hand that the level of socio-economic development may affect the morbidity of acne vulgaris by influencing lifestyle, and on the other hand, reflecting an increase in health awareness and the diagnosis of acne vulgaris.

There are some limitations of this study. On the one hand, since GBD 2019 statistics only cover the national and regional levels, further analysis of acne vulgaris-related data by the province in China is not possible to further clarify the differences in the burden of acne vulgaris between different regions of China. on the other hand, the data analyzed are all from the GBD, as some areas and populations are not very aware of the need to visit the clinic and there are bound to be cases that are not counted.

Acne vulgaris poses a growing disease burden for the Chinese population. Understanding the burden of acne has the potential to reveal the underlying pathogenesis, risk factors, and possible links to comorbidities such as underlying endocrine disorders. First, the potential risk factors for acne are not well understood. Future research could provide some clues to the etiology of acne through statistical analysis of the prevalence of acne in neighboring areas of China, and thus develop health policies for the prevention and early management of acne, to prevent the financial burden of follow-up treatment due to serious sequelae such as scarring. Institutions should focus on the psychological burden when assessing the specific harms of acne-related comorbidities, and doctors should consider the need for psychosocial support and psychological intervention for some patients when seeing them. Thirdly, pharmacoeconomic data on acne treatment are very limited and further pharmacoeconomic studies could help to maximize the use of healthcare resources for the treatment of acne. Fourth, indications for the use of oral antibiotics in acne treatment modalities should be mastered, tailored training and research have been embedded in hospitals to guide clinical practice and minimize skill and knowledge gaps in healthcare professionals actively exploring their epidemiological patterns and their impact on social development are important in guiding health policymaking, improving population health, and effectively diagnosing and preventing disease progression.

## Conclusion

Acne vulgaris poses a serious disease burden on the Chinese population. Actively exploring its epidemiological patterns and impacts on social development is important in guiding health policymaking, improving population health and effectively diagnosing and preventing disease progression.

## Data availability statement

The raw data supporting the conclusions of this article will be made available by the authors, without undue reservation.

## Ethics statement

All protocols were approved by the Institutional Review Board of Health Science Center of Xi’an Jiaotong University. The patients/participants provided their written informed consent to participate in this study.

## Author contributions

YZ: conceptualization and project administration. YW: data curation. YW, YZ, JR, and SX: formal analysis and writing—review and editing. YZ and YW: methodology, visualization, and writing—original draft. All authors contributed to the article and approved the submitted version.

## Footnotes

1
http://ghdx.healthdata.org/

2
http://ghdx.healthdata.org/gbd-results-tool
